# Myocardial T1 measurement in patients with thalassemia major

**DOI:** 10.1186/1532-429X-11-S1-P158

**Published:** 2009-01-28

**Authors:** Taigang He, Maria Paes, John-Paul Carpenter, Andreas Greiser, Daniel Messroghli, Sanjay Prasad, Dudley Pennell, David Firmin

**Affiliations:** 1 CMR Unit, Royal Brompton Hospital and Imperail College London, London, UK; 2 Siemens Medical Solutions, Erlangen, Germany; 3 Cardiac MRI Unit, Franz-Volhard-Klinik Charité, Berlin, Germany

**Keywords:** Cardiovascular Magnetic Resonance, Iron Overload, Thalassemia Major, Myocardial Iron, Cardiac Iron

## Introduction

Cardiac complications secondary to iron overload are the leading cause of death in thalassemia major (TM). Cardiovascular magnetic resonance (CMR) can provide a noninvasive means of measuring the amount of tissue iron. With CMR, tissue iron is detected indirectly by the effect on relaxation times of hydrogen nuclei in the presence of ferritin and hemosiderin iron. The iron results in shortening of proton relaxation times and both CMR T2* and T2 have been validated as non-invasive means for assessment of myocardial iron overload. [1–4]. We have demonstrated a linear correlation between T2 and T2* in patients with iron overload [5]. However, there is currently little data on myocardial iron assessment using the longitudinal relaxation time T1 mainly due to the cardiac and respiratory problems in CMR.

A modified Look-Locker Inversion recovery (MOLLI) has been recently proposed for myocardial T1 quantification [6]. It is of interest, therefore, to investigate whether T1 measurement is affected by iron overload in TM patients. For quantitatively analysis, it is important to further investigate the relationship between T1 and the more established T2* measurements in TM patient population.

In this study therefore, we aimed to compare myocardial T1 [6] and T2* measurements [3]*in vivo* in order to establish the relationship between them. We hypothesised that T1 would correlate with T2* linearly when iron becomes dominant in the myocardium.

## Methods

**Figure 1:**
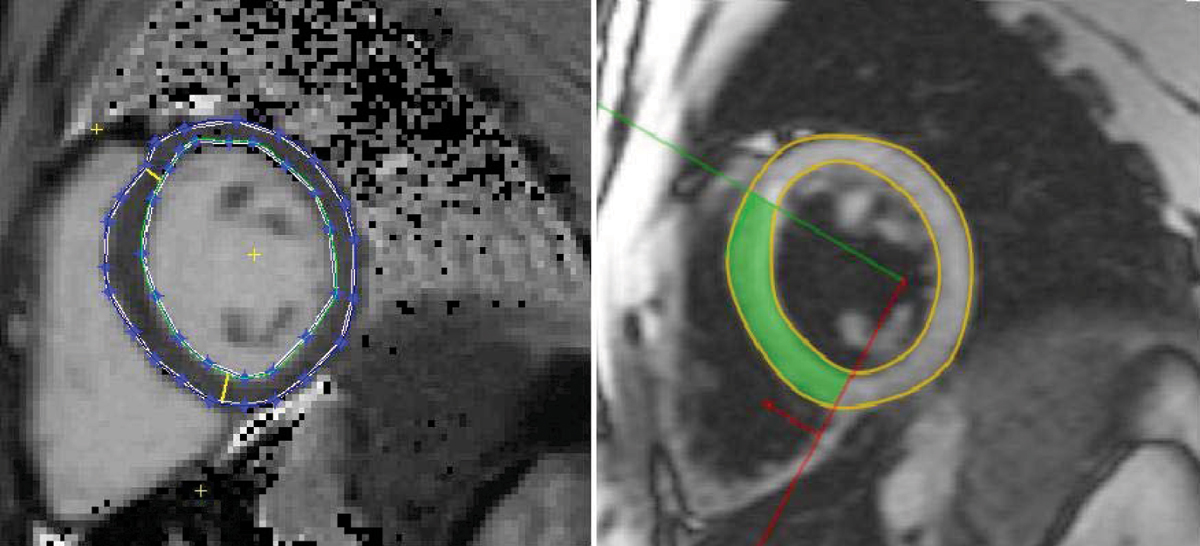
**Drawing of ROIs in the left ventricular septum from the same subject**. *Left*: T1 mapping of the myocardium; *Right*: Black blood T2 image at TE of 4.8 ms.

43 TM patients (age 34 ± 24 years old, 25 males) were studied on a 1.5 T MRI scanner (Siemens Sonata) using a cardiac phased array coil and with ECG gating. All patients were scanned using the black blood T2* [3] sequence and the T1 sequence [6] subsequently, each within a breath-hold. A single mid-ventricular short axis slice was imaged with both T2* and T1 measured in the left ventricular septum (Figure 1). The mono-exponential decay model and the nonlinear curve fitting algorithm were employed for the T2* analysis (CMRtools, Imperial College London). T1 quantification was first acquired using MRMAP http://www.cmr-berlin.org/forschung/mrmapengl/index.html and the septal analysis was done by use of a self-developed software in Matlab.

## Results

**Figure 2:**
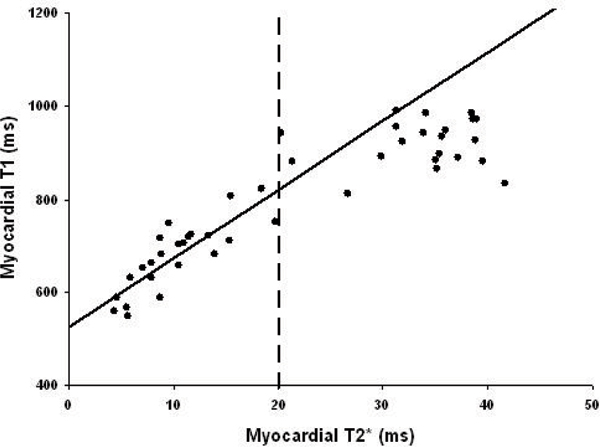
Correlation curve between T1 and T2* measurements. The vertical broken line represents the cut-off myocardial T2* value to distinguish abnormal and normal patients. All data of T2* ≥ 20 ms is fitted with a linear trend line (R = 0.84).

Figure 1 shows a T2 map (left) and a T2* (right) image from the same patient. Figure 2 shows the correlation curve between T1 and T2* drawn from 43 TM patients. There is a linear correlation (R = 0.84) between myocardial T1 and T2* measurements in the septum in iron overloaded patients (T2* ≤ 20 ms). In the normal group of T2* > 20 ms, however, no clear correlation between these two measurements was found.

## Conclusion

T2* of 20 ms is a useful indicator to identify patient with cardiac iron. Myocardial T1 correlated linearly with T2* in TM patients with cardiac iron overload, which is analogous to our previous finding regarding the relationship between T2 and T2* [5]. This study suggests myocardial T1 may potentially be used for assessing iron overload in the heart for TM and other transfusion dependent patients.
